# Stability of class II correction with the Austro Repositioner associated with multi-brackets fixed appliances in dolichofacial patients

**DOI:** 10.1186/s12903-023-03692-7

**Published:** 2024-01-08

**Authors:** María Dolores Austro-Martinez, Ana I. Nicolás-Silvente, Mª Angeles Requena, Marta Carazo-Austro, José Antonio Alarcón

**Affiliations:** 1 Department of Restorative Dentistry, School of Dentistry, CEIR Campus Mare Nostrum, University of Murcia, Murcia, 30008 Spain; 2 Department of Dental Pathology and Therapeutics, School of Dentistry, CEIR Campus Mare Nostrum, University of Murcia, Murcia, 30008 Spain; 3 Private practice in orthodontics, Málaga, 29010 Spain; 4 Undergraduate student, Faculty of Odontology, University of Granada, Granada, 18071 Spain; 5 Department of Stomatology, Section of Orthodontics, Faculty of Odontology, University of Granada, Granada, 18071 Spain

**Keywords:** Class II, Mandibular retrognathism, Dolichofacial pattern, Fixed functional appliance, Austro Repositioner, Stability

## Abstract

**Background:**

The purposes of the present study were to evaluate the changes produced by the Austro Repositioner, and to assess the stability of Class II malocclusion treatment with the Austro Repositioner associated with fixed appliances and its capacity to control the vertical dimension in dolichofacial patients.

**Methods:**

A group of patients with Class II malocclusion due to mandibular retrognathism and a dolichofacial growth pattern treated with the Austro Repositioner combined with fixed appliances were compared to a matched untreated control group of subjects with Class II malocclusion. Evaluations were made on the basis of lateral cephalograms taken at T1 (initial records), T2 (end of treatment), and T3 (1 year after treatment). Statistical comparisons were performed with paired- and two-sample t tests.

**Results:**

The experimental (treated) group comprised 30 patients, 14 boys and 16 girls, and the control group comprised 30 subjects (15 boys and 15 girls) with similar ages at T1, T2 and T3.

In the treated group, a significant decrease in the ANB angle was found (− 3.79 ± 1.46; *p* < 0.001). No significant differences were found in the maxillary skeletal measurements. In contrast, the SNB angle showed a significant increase of 3.77 ± 1.49 in the treated group compared with a nonsignificant increase of 0.77 ± 1.55 in the control group (*p* = 0.002). Vertical changes showed a significant decrease in the FMA angle (− 3.36 ± 1.62), while the lower anterior facial height distance and the overbite increased significantly in the treated group, reflecting a change in vertical dimensions after treatment. No significant changes were observed in either the treated or control group during the one-year posttreatment period; thus, the treatment results remained stable.

**Conclusions:**

The Austro Repositioner combined with fixed appliances could be considered an optimal treatment modality in Class II dolichofacial patients.

## Background

The success of treating Class II malocclusion with mandibular hypoplasia through functional appliances depends, among other factors, on the patient’s vertical facial pattern. In general, functional devices produce posterior mandibular rotation, increase the mandibular plane angle (MPA), increase the lower anterior facial height, and open the gonial angle [1–10]. This is a limitation in dolichofacial subjects, as there is a poor response to mandibular advancement. Clockwise rotation of the mandible is due to an unwanted effect of molar extrusion, which is not sufficiently compensated for by posterior facial growth [11].

Therefore, functional appliances to treat skeletal Class II patients with mandibular retrusion and a dolichofacial growth pattern should be able to control the vertical growth component to prevent clockwise rotation of the mandible, avoid an increase in the MPA, and guide mandibular growth in an anterior rather than a vertical direction. Some studies have shown adequate vertical control during functional treatment, including treatment with a twin block [12], clear aligner [12] Jasper Jumper [13], Herbst device [14], Beek headgear-activator [15], and Austro Repositioner [16]; nevertheless, there is a lack of agreement in the literature.

Vertical control is achieved by avoiding the extrusion of molars and even producing their intrusion, resulting in mandibular antero-rotation and, therefore, a more significant sagittal effect on Class II malocclusion.

A previous study using the Austro Repositioner found positive short-term results when used in Class II dolichofacial patients [16]. This study showed significant improvements in Class II patients and adequate vertical control, with a decreased MPA and counterclockwise mandibular rotation. However, there is a lack of studies evaluating the long-term effects of functional devices on these patients.

Therefore, the objectives of this study were, firstly, to evaluate the changes produced by the Austro Repositioner, and secondly to assess the stability of Class II malocclusion treatment with the Austro Repositioner associated with fixed appliances and its capacity to control vertical dimensions in dolichofacial patients.

## Material and methods

A retrospective clinical study was designed. Subjects were evaluated three times: before treatment (T1); after functional orthopedic and fixed appliances treatment (T2); and 1 year posttreatment (T3).

Two study groups were established

- The experimental group was composed of patients with skeletal Class II malocclusion due to mandibular retrognathism and a dolichofacial growth pattern, consecutively recruited if they met the eligibility criteria, from a private practice and treated by the same orthodontist (M.D.A-M).
- The control group was composed of patients with similar characteristics to those in the experimental group but who were not treated and were selected from the online Craniofacial Legacy Collection (http://www.aaoflegacycollection.org), which consists of different growth studies known as the Michigan and Burlington studies.

G Power software was used to calculate the sample size, revealing that at least 23 patients were required per group to detect a mean difference (effect size) of 1.2° and 1.1 mm (α = 0.05, and 1 − β = 0.90), based on a previous study [16].

The study was conducted according to the guidelines of the Declaration of Helsinki and approved by the Ethics Committee of University of Murcia (approved on 29 June 2015). Written informed consent was provided by the subjects and their parents/guardians of all the patients.

### Inclusion criteria

At the start of treatment, all selected subjects presented skeletal Class II malocclusion (ANB angle > 5°) resulting from mandibular retrognathia (SNB angle < 78°), a dolichofacial growth pattern (FMA angle > 28°), overjet (OJ) ≥ 5 mm, a symmetrical molar Class II relationship (minimum severity of a quarter of a Class II molar relationship; the mean amount of a Class II molar relationship was 5.7 ± 2.03 mm) and skeletal maturation between the CS3 and CS4 stages, which is when the growth peaks occur, according to the cervical vertebral maturation (CVM) method [17].

### Exclusion criteria

Patients with premolar extractions, agenesis of permanent teeth, severe facial asymmetry determined by radiographic and clinical examination, congenital syndromes, posterior crossbites or transverse deficiencies, TMJ disorders, previous orthopedic/orthodontic treatment, and poor oral hygiene were excluded from the study.

### Treatment protocol for class II malocclusion

All patients were treated in two phases. Patients wore an Austro Repositioner, specially designed for dolichofacial patients, as a fixed functional appliance during the first phase. This appliance has an acrylic wedge located in the palatal ridge area. Two 0.9-mm steel bars stem from this wedge and are anchored in bands around the first permanent upper molars. The acrylic wedge has an inclined plane. Due to this design, the lower incisors contact the most posterior area of the acrylic wedge, the thickest area, sliding down the inclined plane to a more anterior position and bite in front of the thinnest area attached to the maxillary incisors.

**Fig. 1:**
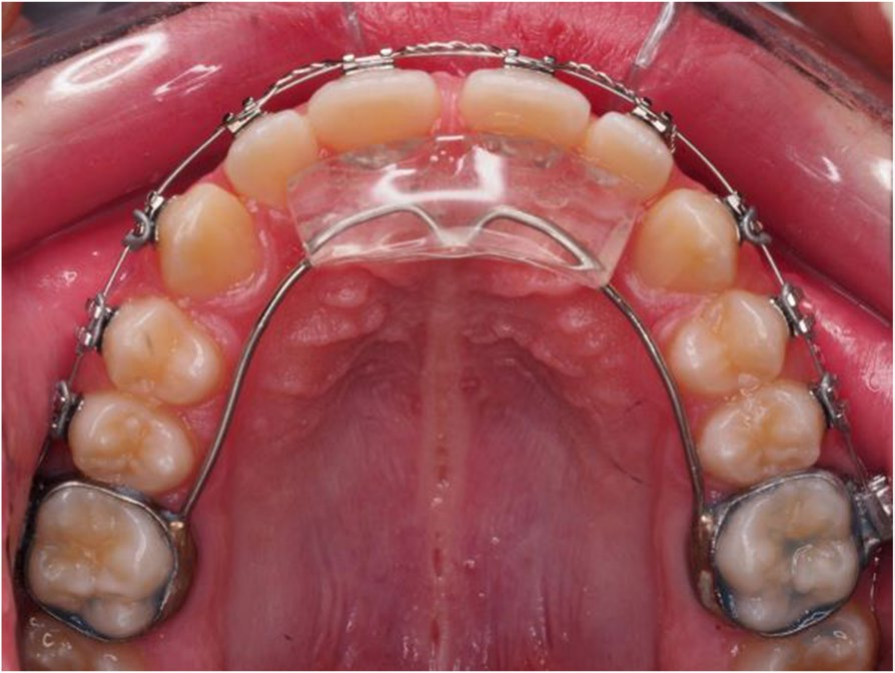
Austro Repositioner designed for dolichofacial patients, combined with multibracket fixed appliances

In this design, when the mandible is closed, the lower incisors are located in front of the acrylic wedge and remain between the acrylic and the maxillary incisors. This leads to contact between the posterior sectors, allowing the patient to bite during the treatment time with his molars without producing vertical changes such as molar extrusion. In addition, the design allows the combined use of other fixed appliances, such as braces (Fig. 1). Bite registration was made according to a previous study [16].

After a minimum period of 1 year of functional treatment, orthodontic treatment continued with a simultaneous second phase with multibracket fixed appliances (Hilgers Edgewise Bracket System, conventionally ligated, 0.022-in. slot; Ormco, Glendora, California). Class II elastics were not used during the multibracket phase. Treatment continued with both devices simultaneously for 24–30 months, followed by a 12-month retention period with a standard removable Hawley retainer (6 months full-time, 6 months at night) in the upper arch and a fixed retainer from canine to canine in the lower arch.

### Measurement method

In all subjects, lateral cephalometric radiographs were taken with the teeth in centric occlusion and the head oriented with the plane of Frankfort horizontal, according to a previously described protocol [16]. The following linear and angular measurements were made as described by Steiner [18], Ricketts [19], and McNamara [20]: SNA (°), SNB (°), ANB (°), Pt A-Na perp (mm), Pg-Na perp (mm), Co-Pg (mm), FMA (°), LAFH (mm), overbite (OB) (mm), OJ (mm), U1 to SN (°), L1 to GoMe (°), and interincisal angle (°).

Measurements were carried out with Dolphin Imaging software 11.0 (Chatsworth, CA, USA).

### Statistical analysis

A standard statistical software package (IBM SPSS statistics 20, IBM Armonk, New York, NY, USA) was used for statistical analysis. After confirming the normal distribution of the variables, differences in pretreatment variables between the groups were determined by two-sample t tests. Changes within the groups were assessed using paired-sample t tests, comparing values between T0, T1, and T2. Comparisons of changes between the two groups were made using two-sample t tests.

All images were evaluated by a single experienced observer (M.D.A.-M). To determine the reliability of the measurements, the same observer re-evaluated 30 randomly selected images, and another independent expert (J.A.A.) also evaluated them. Inter- and interrater agreements were calculated using Cohen’s kappa (ƙ) coefficient [21].

## Results

The experimental (treated) group comprised 30 patients, 14 boys and 16 girls, with a mean age of 11.5 years at T1, 14.7 years at T2, and 15.8 years at T3, and the control group comprised 30 subjects (15 boys and 15 girls, with a mean age of 11.7 years at T1, 14.3 years at T2, and 15.4 years at T3).

The inter- and intrarater agreement coefficients were ƙ = 0.89 and ƙ = 0.91, respectively.

There were no statistically significant differences between the treated and control groups at T1 (Table 1).

**Table 1:** Intergroup comparison at the beginning (T1)

T1	Treated group		Control group		*p*-value
	Mean	SD	Mean	SD	
**Age**	11.82	1.20	11.78	0.61	0.924
**SNA (°)**	81.10	2.73	81.13	2.37	0.873
**SNB (°)**	74.83	2.84	74.89	2.16	0.579
**ANB (°)**	6.27	1.36	6.44	0.83	0.631
**Pt A-Na perp (mm)**	2.76	1.53	2.64	1.21	0.398
**Pg-Na perp (mm)**	−2.68	2.29	−2.73	2.27	0.672
**Co-Pg (mm)**	100.29	4.66	100.24	4.57	0.943
**FMA (°)**	29.80	2.51	29.85	2.18	0.545
**LAFH (mm)**	57.03	1.62	61.62	2.88	0.497
**OB (mm)**	2.20	0.44	2.16	0.67	0.731
**OJ (mm)**	6.75	1.42	6.71	0.82	0.857
**U1 to SN (°)**	103.16	2.44	103.08	1.31	0.356
**L1 to GoMe (°)**	93.18	4.13	93.15	3.86	0.298
**Interincisal Angle(°)**	127.18	5.26	127.25	1.56	0.933

Table 2 shows inter- and intragroup comparisons of differences between the treatment and observation periods (T2–T1). In the treated group, a significant decrease in the ANB angle was found (− 3.79 ± 1.46; *p* < 0.001). No significant differences were found in the maxillary skeletal measurements. In contrast, the SNB angle showed a significant increase of 3.77 ± 1.49 in the treated group compared with a nonsignificant increase of 0.77 ± 1.55 in the control group (*p* = 0.002).

**Table 2:** Inter and intra-groups comparison of the treatment/observation period time (T2-T1)

T2-T1	Treated group			Control group			Treated vs. Control
Measurements	Mean differences	SD	*p* Value	Mean differences	SD	*p* Value	*p* Value
**SNA (°)**	−0.12	0.61	0.937	0.04	1.66	0.732	0.693
**SNB (°)**	3.77	1.49	< 0.001	0.77	1.55	< 0.001	< 0.001
**ANB (°)**	−3.79	1.46	< 0.001	−0.78	0.96	< 0.001	< 0.001
**Pt A-Na perp (mm)**	0.17	0.69	0.604	0.33	1.52	0.351	0.325
**Pg-Na perp (mm)**	−3.70	1.64	< 0.001	−0.91	2.38	< 0.001	< 0.001
**Co-Pg (mm)**	8.50	2.99	< 0.001	2.55	1.55	< 0.001	< 0.001
**FMA (°)**	−3.36	1.62	< 0.001	0.70	1.70	< 0.001	< 0.001
**LAFH (mm)**	3.32	1.78	< 0.001	0.64	1.69	< 0.001	< 0.001
**OB (mm)**	0.60	0.59	< 0.001	0.11	1.10	< 0.001	< 0.001
**OJ (mm)**	−3.72	1.19	< 0.001	−0.21	1.34	< 0.001	< 0.001
**U1 to SN (°)**	−2.48	2.56	< 0.001	−0.52	1.61	< 0.001	< 0.001
**L1 to GoMe (°)**	−2.68	3.70	< 0.001	0.42	1.08	< 0.001	< 0.001
**Interincisal Angle (°)**	4.29	5.29	< 0.001	−0.47	3.78	< 0.001	< 0.001

No significant differences were observed in the maxillary cephalometric measurements (SNA angle and Pt A-Na perp). In contrast, the SNB angle, Pg-Na perp, and Co-Pg distance increased significantly in the treated group compared to the control group.

Vertical changes showed a significant decrease in the FMA angle (− 3.36 ± 1.62), while the LAFH distance and the OB increased significantly in the treated group, reflecting a change in vertical dimensions after treatment with an Austro Repositioner in dolichofacial Class II patients.

Finally, retroclination of both the upper and lower incisors was found after treatment.

Table 3 shows intra- and intergroup comparisons of differences between the posttreatment and 1-year posttreatment/observation periods (T3-T2). No significant changes were observed in either the treated or control group during the posttreatment period. Intergroup comparisons showed the same significant differences observed during the treatment/observation period (T2-T1).

**Table 3:** Intra- and inter-groups during posttreatment and 1-year post-treatment/observation period (T3-T2)

T3-T2	Treated Group			Control Group			Treated vs Control
	Mean differences	SD	*p* Value	Mean differences	SD	*p* Value	*p* Value
**SNA (°)**	0.5	1.33	0.735	0.24	1.45	0.321	0.493
**SNB (°)**	0.14	2.24	0.342	0.65	1.32	0.143	< 0.001
**ANB (°)**	−0.67	0.39	0.823	−0.14	1.61	0.235	< 0.001
**Pt A-Na perp (mm)**	0.23	1.33	0.643	0.04	1.40	0.064	0.323
**Pg-Na perp (mm)**	0.69	2.11	0.321	0.75	2.33	0.115	0.022
**Co-Pg (mm)**	0.76	1.87	0.533	0.73	1.42	0.167	0.001
**FMA (°)**	0.57	1.38	0.241	0.84	1.68	0.311	0.013
**LAFH (mm)**	0.21	2.31	0.265	0.36	1.49	0.121	< 0.001
**OB (mm)**	0.35	1.16	0.123	0.24	2.09	0.067	< 0.001
**OJ (mm)**	0.85	1.21	0.357	0.79	2.27	0.077	< 0.001
**U1 to SN (°)**	−0.05	2.27	0.467	0.36	1.11	0.113	0.031
**L1 to GoMe (°)**	−0.03	1.27	0.153	0.54	1.36	0.081	0.015
**Interincisive Angle (°)**	0.17	3.16	0.137	−0.15	2.42	0.061	0.001

**Fig. 2:**
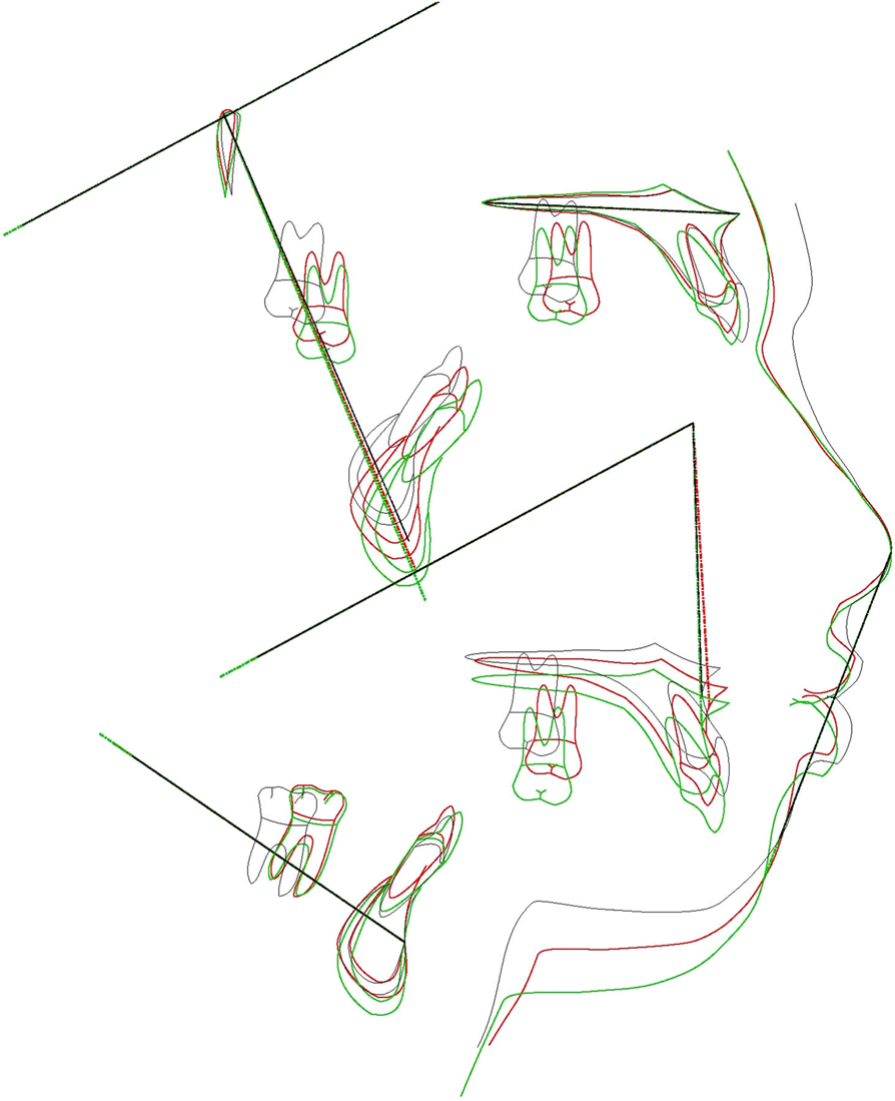
Cephalometric superimposition of pre-treatment (black), post-treatment (red), and 1 year follow up (green)

Figure 2 shows cephalometric superimposition of post and pre-treatment changes as well as 1 year follow up.

## Discussion

This study evaluated the the changes produced by the Austro Repositioner, and the stability of the results of treatment with the Austro Repositioner as a fixed functional appliance combined with fixed multibracket appliances in patients with skeletal Class II malocclusion, mandibular retrognathism and a dolichofacial growth pattern.

After active treatment (T2-T1), there was a significant improvement in skeletal Class II malocclusion due exclusively to changes in the mandible. The mandible increased in size and was positioned more anteriorly due to counterclockwise rotation. Importantly, contrary to the control group, vertical control of the dolichofacial pattern was achieved in the treated group, as demonstrated by a significant decrease in the FMA angle, reflecting the counterclockwise rotation mentioned above. At the dental level, the OJ was reduced due to the improvement in the maxillo-mandibular sagittal skeletal relationship, as well as the retroclination of both the upper and lower incisors. These changes in upper and lower incisor inclination, together with the reduction in the FMA angle, contributed to the significant increase in OB.

One year posttreatment (T3-T2), no significant changes were observed in any of the cephalometric variables analyzed in either group, while the same significant differences observed between the two groups after the treatment period (T2-T1) were maintained 1 year later (T3-T2). The two variables pertaining to the maxilla (SNA angle and Pt A-Na perp) continued to show no significant differences between the two groups. These results indicate that 1 year later, the results achieved by the treatment remained stable. Therefore, we can consider that treatment with the Austro Repositioner combined with fixed appliances is effective for the treatment of skeletal Class II malocclusion with mandibular retrognathia in patients with a dolichofacial growth pattern.

The results confirm previous findings on the effects of the Austro Repositioner in the short term [16], highlighting the good vertical control that can be achieved; thus, it could be recommended in dolichofacial patients.

The favorable results found in the study can be attributed, among other factors, to the special design of the device, which causes a progressive advancement of the mandible while maintaining the posterior occlusal contacts at all times and thus avoiding the extrusion of posterior teeth and worsening of the vertical pattern. The timing chosen for treatment, between the CS3 and CS4 stages of skeletal maturation [17, 22], may also have favored these good results [23, 24].

According to the literature, there have been few studies specifically on the stability of treatment with functional appliances in dolichofacial patients [14].

One of the findings of greatest clinical interest of our study is the large increase in the SNB angle observed after treatment, which remained stable after 1 year of follow-up. Other studies have also found significant but smaller increases in the SNB angle in dolichofacial patients treated with fixed functional appliances [25–28].

Similarly, the ANB angle was significantly reduced in the treated group, and no significant changes were found during the year after treatment, reflecting the stability of the improvement in skeletal Class II malocclusion achieved with this treatment protocol. The reduction in the ANB angle observed in our study is much higher than that obtained in other studies with different functional devices, such as the Herbst device [27, 28] or the twin block device [29, 30].

In reference to the SNA angle, no significant changes were observed, indicating that the Austro Repositioner had no effects at the maxillary level.

Both the Pg position (Pg-Na perp) and total mandibular length (Co-Pg) significantly increased in our study, again indicating the effect of treatment on mandibular growth. Other authors have also observed increases in these measurements, but smaller increases than those observed in our study [29–32].

One of the main objectives of our study was to evaluate the effect of the Austro Repositioner on vertical dimensions in dolichofacial patients. The MP-SN and FMP angles decreased, while the OB increased significantly; there was counterclockwise rotation, which is a relevant finding since most functional appliances produce opening of the facial axis, an increase in the MP-SN angle, and clockwise mandibular rotation [33, 34].

In the treatment of skeletal Class II malocclusion, the MP-SN angle can be controlled if the posterior teeth are not extruded during the process [25, 35]; this is the mechanism of action by which the Austro Repositioner achieves vertical control, even favoring counterclockwise rotation of the mandible. These results remained stable 1 year posttreatment. The ability to control vertical dimensions is more limited with other fixed functional appliances, with which significant changes in the vertical pattern have also been described [27, 36–38].

At the dental level, in our study, there was a retroclination of both the upper and lower incisors, but no changes were observed in the position of the lower incisors 1 year after treatment, indicating stability of the OJ correction due to the mandibular skeletal effect. The mandibular increment observed during treatment remained stable 1 year later; thus, there were no changes in the position of the incisors that could have masked this skeletal effect. These data show that the skeletal changes observed in the mandible played an essential role in reducing the OJ in the patients treated with the Austro Repositioner.

The most common dental effect found in the literature is the retroclination of the upper incisors and the proclination of the lower incisors and, consequently, a reduction of the OJ by dentoalveolar changes after treatment with functional appliances [29, 34, 37, 39, 40]. There have been very few studies on stability of incisor changes following treatment with functional appliances in dolichofacial patients, which precludes comparisons with our results.

Its ability to improve skeletal Class II malocclusion, as well as its ability to modify the vertical pattern, at least 1 year after treatment, leads us to consider the Austro Repositioner as an effective option for the treatment of Class II skeletal malocclusion of mandibular origin in patients with a dolichofacial growth pattern.

The major limitation of this study is its retrospective nature; as such, patients could not be randomly assigned to a particular treatment protocol and evaluated prospectively. The decision regarding the modality of treatment for skeletal Class II malocclusion was made largely based on the clinician’s preferences.

Future long-term studies with larger samples are needed to confirm these results.

## Conclusions

The Austro Repositioner combined with fixed appliances could be considered an optimal treatment modality in Class II dolichofacial patients. Favorable changes of Class II correction remained stable 1 year posttreatment, and adequate vertical control was achieved.

## Data Availability

The data underlying this article are available in the article and available from the corresponding author on reasonable request.
